# Clinically Relevant Four-Level Cancer-Related Fatigue Among Patients With Various Types of Cancer

**DOI:** 10.6004/jadpro.2016.7.1.2

**Published:** 2016-01-01

**Authors:** Hsiao-Lan Wang,1, Ming Ji,1, Connie Visovsky,1, Carmen S. Rodriguez,1, Amanda F. Elliott,1, Clement K. Gwede,2, Tapan A. Padhya,2,3, Marion B. Ridley,3,4, Susan C. McMillan,1

**Affiliations:** 1College of Nursing University of South Florida, Tampa, Florida; 2Moffitt Cancer Center, Tampa, Florida; 3James A. Haley VAMC, Tampa, Florida; 4University of South Florida, Tampa, Florida

**Clinically Relevant Four-Level Cancer-Related Fatigue Among Patients With Various Types of Cancer**

A continuing education article for nurse practitioners, physician assistants, clinical nurse specialists, advanced degree nurses, oncology and hematology nurses, pharmacists, and physicians.

**Release date:** January 15, 2016

**Expiration date:** January 15, 2017

**Expected time to complete this activity as designed:** 1.0 hours

**Meniscus Educational Institute**

3131 Princeton Pike,

Building 1, Suite 205A

Lawrenceville, NJ 08648

Voice: 609-246-5000

Fax: 609-449-7969

E-mail: lrubin@meniscusedu.com

**Journal of the Advanced Practitioner in Oncology**

94 N. Woodhull Road

Huntington, NY 11743

Voice: 631-692-0800

Fax: 631-692-0805

E-mail: claudine@harborsidepress.com

© *2016, Meniscus Educational Institute. All rights reserved.*

## Faculty

**Hsiao-Lan Wang, PhD, RN, CMSRN, HFS,** College of Nursing University of South Florida

**Ming Ji, PhD,** College of Nursing University of South Florida

**Connie Visovsky, PhD, RN, ACNP-BC,** College of Nursing University of South Florida

**Carmen S. Rodriguez, PhD, RN, ACNP-BC, AOCN®,** College of Nursing University of South Florida

**Amanda F. Elliott, PhD, RN, ARNP,** College of Nursing University of South Florida

**Clement K. Gwede, PhD, RN, MPH,** Division of Cancer Prevention and Control, Moffitt Cancer Center

**Tapan A. Padhya, MD,** College of Medicine Otolaryngology at the University of South Florida, Moffitt Cancer Center, and James A. Haley VAMC

**Marion B. Ridley, MD,** College of Medicine Otolaryngology at the University of South Florida and James A. Haley VAMC

**Susan C. McMillan, PhD, RN, ARNP, FAAN,** College of Nursing University of South Florida

## Activity Rationale and Purpose

Symptom management, including the management of cancer-related fatigue, is a primary role of advanced practitioners (APs) specializing in oncology. Analyzing and/or translating research evidence into practice is essential in improving patient-centered outcomes. Translational research to improve clinicians’ abilities to screen, assess, and intervene to reduce CRF is of high priority to the National Cancer Institute and the Oncology Nursing Society. However, fatigue is very subjective, and current research design issues make it difficult for APs to identify and utilize evidence-based assessment tools and guidelines. The purpose of this article is to act as a guide for APs on how to use the tools and guidelines to accurately define severity of CRF, recognize patient variables that may be barriers in preventing and/or treating CRF, and initiate effective interventions and patient education.

## Intended Audience

The activity’s target audience will consist of nurse practitioners, physician assistants, clinical nurse specialists, advanced degree nurses, oncology and hematology nurses, pharmacists, and physicians.

## Learning Objectives

After completing this educational activity, participants should be able to:

- Analyze strengths and weaknesses of currently available evidence-based research in the area of cancer-related fatigue (CRF)
- Describe how assessment tools and guidelines can be translated into clinical practice and help to overcome barriers at the patient, professional and system level
- Apply existing evidence-based tools and guidelines in defining severity of CRF
- Recognize individual patient variables that may be barriers to prevention and/or treatment of CRF and develop more individualized and effective CRF treatment plans, patient education, and counseling

## Continuing Education

**Statement of Credit—Participants who successfully complete this activity (including the submission of the post-test and evaluation form) will receive a statement of credit.**

**Physicians.** The Meniscus Educational Institute is accredited by the Accreditation Council for Continuing Medical Education (ACCME) to provide continuing medical education for physicians.

The Meniscus Educational Institute designates this journal article for a maximum of 1.0 *AMA PRA Category 1 Credits*™. Physicians should claim only the credit commensurate with the extent of their participation in the activity.

**Nurses.** This activity for 1.0 contact hour is provided by the Meniscus Educational Institute.

The Meniscus Educational Institute is accredited as a provider of continuing nursing education by the American Nurses Credentialing Center’s Commission on Accreditation.

Provider approved by the California Board of Registered Nursing, Provider No. 13164, for 1.0 contact hour.

**Pharmacists.** The knowledge-based accredited education lectures are intended for pharmacists involved in the care of cancer patients. This educational activity is sponsored by the Meniscus Educational Institute.

The Meniscus Educational Institute is accredited by the Accreditation Council for Pharmacy Education (ACPE) as a provider of continuing pharmacy education. The ACPE Universal Activity Number assigned to this program, for 1.0 contact hour, is 0429-9999-15-017-H01-P.

## Financial Disclosures

All individuals in positions to control the content of this program (eg, planners, faculty, content reviewers) are expected to disclose all financial relationships with commercial interests that may have a direct bearing on the subject matter of this continuing education activity. Meniscus Educational Institute has identified and resolved all conflicts of interest in accordance with the MEI policies and procedures. Participants have the responsibility to assess the impact (if any) of the disclosed information on the educational value of the activity.

**Faculty**

**Hsiao-Lan Wang, PhD, RN, CMSRN, HFS,** has nothing to disclose.

**Ming Ji, PhD,** has nothing to disclose.

**Connie Visovsky, PhD, RN, ACNP-BC,** has nothing to disclose.

**Carmen S. Rodriguez, PhD, RN, ACNP-BC, AOCN®,** has nothing to disclose.

**Amanda F. Elliott, PhD, RN, ARNP,** has nothing to disclose.

**Clement K. Gwede, PhD, RN, MPH,** has nothing to disclose.

**Tapan A. Padhya, MD,** has nothing to disclose.

**Marion B. Ridley, MD,** has nothing to disclose.

**Susan C. McMillan, PhD, RN, ARNP, FAAN,** has nothing to disclose.

**Lead Nurse Planner**

**Wendy J. Smith, ACNP, AOCN®,** has nothing to disclose.

**Planners**

**Jeannine Coronna** has nothing to disclose.

**Claudine Kiffer** has nothing to disclose.

**Terry Logan, CHCP,** has nothing to disclose.

**Pamela Hallquist Viale, RN, MS, CNS, ANP,** has nothing to disclose.

**Lynn Rubin** has nothing to disclose.

**Content Reviewers**

**Glenn Bingle, MD, PhD, FACP,** has nothing to disclose.

**Kate D. Jeffers, PharmD, BCOP,** has nothing to disclose.

**Karen Abbas, MS, RN, AOCN®,** has nothing to disclose.

**Wendy J. Smith, ACNP, AOCN®,** has nothing to disclose.

## Disclaimer

This activity has been designed to provide continuing education that is focused on specific objectives. In selecting educational activities, clinicians should pay special attention to the relevance of those objectives and the application to their particular needs. The intent of all Meniscus Educational Institute educational opportunities is to provide learning that will improve patient care. Clinicians are encouraged to reflect on this activity and its applicability to their own patient population.

The opinions expressed in this activity are those of the faculty and reviewers and do not represent an endorsement by Meniscus Educational Institute of any specific therapeutics or approaches to diagnosis or patient management.

## Product Disclosure

This educational activity may contain discussion of published as well as investigational uses of agents that are not approved by the US Food and Drug Administration. For additional information about approved uses, including approved indications, contraindications, and warnings, please refer to the prescribing information for each product.

## How to Earn Credit

To access the learning assessment and evaluation form online, visit www.meniscusce.com

**Statement of Credit:** Participants who successfully complete this activity (including scoring of a minimum of 70% on the learning assessment and complete and submit the evaluation form with an E-mail address) will be able to download a statement of credit.

## ABSTRACT

The purpose of this study was to identify the association between clinically relevant four-level cancer-related fatigue (CRF) and quality of life (QOL). This secondary data analysis included 152 participants who completed both the 0 to 10 fatigue scale and the Multidimensional Quality of Life Scale-Cancer (MQOL-C). The four-level CRF included no CRF (score: 0), mild CRF (scores: 1–3), moderate CRF (scores: 4–6), and severe CRF (scores: 7–10). The MQOL-C contains five domains. Multiple linear regression models and post hoc analyses were applied while controlling for age, gender, education, marital status, racial background, cancer type, and time after diagnosis. Participants in the less severe CRF subgroup had significantly better scores on total QOL and QOL domains, except for the symptom distress domain. Significant between-CRF-level differences were only found in some of the QOL score comparisons. No difference between mild and moderate CRF subgroups was found in total QOL or in interpersonal well-being (no CRF > mild CRF, moderate CRF > severe CRF). There was no difference between the no and mild CRF subgroups in physical well-being (no CRF, mild CRF > moderate CRF > severe CRF). Our findings support the importance of using clinical guidelines to screen, evaluate, and manage CRF.

## ARTICLE

Cancer-related fatigue (CRF) was identified in the 2013 National Oncology Nurse Survey as the symptom that is the most distressing to patients and the most difficult to manage (LoBiondo-Wood et al., 2014). The National Comprehensive Cancer Network (NCCN) conceptually defines CRF as "a distressing, persistent, subjective sense of physical, emotional, and/or cognitive tiredness or exhaustion related to cancer or cancer treatment that is not proportional to recent activity and interferes with usual functioning."Berger et al., 2010). Based on this definition, the NCCN developed Clinical Practice Guidelines with an algorithm to manage CRF (Berger et al., 2015). The first phase in the algorithm is screening for CRF. The NCCN recommends using a semiquantitative assessment to screen for and document CRF if it is present. On a 0 to 10 numeric rating scale, no CRF has a score of 0, mild CRF has a score between 1 and 3, moderate CRF has a score between 4 and 6, and severe CRF has a score between 7 and 10.

## FOUR-LEVEL CATEGORIZATION OF CRF

Use of the four-level categorization (no, mild, moderate, and severe symptom intensity) is clinically relevant. The four-level categorization has been suggested to (1) inform treatment decisions, (2) integrate provider-patient discussion on symptom management, and (3) communicate with stakeholders (lay individuals) in the development of policy and clinical practice guidelines (Anderson, 2005; Wang et al., 2014). Therefore, understanding the relationship between the four-level categorization of CRF (four-level CRF) and clinically meaningful outcomes is important to clinicians who assess CRF in their practice (Berger et al., 2015; Paul, Zelman, Smith, & Miaskowski, 2005).

Gaps exist in the literature investigating the effect of categorized CRF levels on clinical outcomes. First, studies have failed to compare clinical outcomes among different level CRF subgroups (i.e., between-CRF-level differences in clinical outcomes). A study by Mendoza et al. (1999) showed that patients who had severe CRF reported markedly increased fatigue interference. However, this study did not investigate differences in fatigue interference between mild and moderate CRF subgroups. Another study found that cancer patients with moderate/severe CRF were more likely to report poor Eastern Cooperative Oncology Group performance status (ECOG ≥ 1; Wang et al., 2014). Therefore, it is not clear whether moderate and severe CRF were associated with poor performance status.

Second, some studies investigated CRF levels in clinical outcomes only in breast cancer patients (Dirksen, Belyea, & Epstein, 2009; Ho, Fong, & Cheung, 2014; Stover et al., 2013). Third, several studies were conducted in cancer patients of male/female gender, various racial backgrounds, a wide range of ages and years after cancer diagnosis, and patients with diverse cancer types, but without proper statistical control (Hwang, Chang, Cogswell, & Kasimis, 2002; Mendoza et al., 1999; Wang et al., 2014). Males, nonwhite minorities, and those 55 or older, with more than 1 year after cancer diagnosis, or those with lung cancer, reported more severe CRF than their counterparts (Fisch et al., 2014).

## QUALITY OF LIFE

Quality of life (QOL) has been considered an important clinical outcome for cancer treatment efficacy, in addition to survival rate (Thong et al., 2013). The National Cancer Institute (NCI) has suggested using QOL as an endpoint in clinical trials (Clauser, Ganz, Lipscomb, & Reeve, 2007; Lipscomb et al., 2007). Patients with CRF had significantly worse QOL in overall health (Alexander, Minton, Andrews, & Stone, 2009; Murphy, Alexander, & Stone, 2006; Van Belle et al., 2005) and domain-specific well-being (Andrykowski, Donovan, Laronga, & Jacobsen, 2010; Andrykowski, Schmidt, Salsman, Beacham, & Jacobsen, 2005; Sadler et al., 2002). According to the Centers for Disease Control and Prevention, QOL is a multidimensional concept that includes subjective perception of physical and psychological well-being and their correlates (CDC, 2011), such as interpersonal well-being (Edmond et al., 2013; Huang & Hsu, 2013; Kwan et al., 2013), nutrition (Bazzan, Newberg, Cho, & Monti, 2013; Mohammadi, Sulaiman, Koon, Amani, & Hosseini, 2013), and symptoms (Denieffe, Cowman, & Gooney, 2013; Henoch & Lovgren, 2014).

Previous studies describing effects of CRF levels on QOL applied SF-36 (physical and mental components; Stover et al., 2013) and Functional Assessment of Cancer Therapy (FACT-G; physical well-being, functional well-being, emotional well-being, and social well-being) scales (Dirksen et al., 2009; Ho et al., 2014; Hwang et al., 2002).

## STUDY PURPOSE

The purpose of this study was to use the Multidimensional Quality of Life Scale–Cancer (MQOL-C) as the dependent variable to understand the association between the four-level CRF and QOL while controlling for age, gender, education, marital status, racial background, time after diagnosis, and cancer type among cancer patients. The MQOL-C contains five dimensions (i.e., domains): physical well-being, psychological well-being, interpersonal well-being, nutrition domain, and symptom distress domain (Padilla, 1992; Padilla & Grant, 1985; Padilla et al., 1992; Pinar, 2004; Pinar, Salepci, & Afsar, 2003). The MQOL can be investigated by its total score and domain subscores.

We anticipated that our study would provide new information on associations between four-level CRF and QOL. First, we tested the four-level CRF as a categorized predictor of QOL, then investigated the QOL differences among the four CRF level subgroups. Second, we adjusted for multiple demographic and clinical factors among diverse cancer patients across all analyses. Our research questions were the following: (1) Is there an association of the four-level CRF with total QOL and QOL domains after adjusting for age, gender, education, marital status, racial background, cancer type, and time after diagnosis? (2) If there is an association, are there between-CRF-level differences in the total QOL and QOL domains after adjusting for age, gender, education, marital status, racial background, cancer type, and time after diagnosis?

## METHODS

**Sample**

We conducted a secondary data analysis from a cancer symptom study. The design, instruments, and participant characteristics have been presented previously (McMillan, Choe, & Rheingans, 2014). Briefly, the sample for the study consisted of 234 patients with a cancer diagnosis who were treated for cancer or cancer symptoms at a local medical center or at an NCI-designated comprehensive cancer center. Eligible participants were older than 18, able to read and understand English, alert and oriented, and able to consent to the study. Patients who were within 3 weeks following surgery were excluded so as to avoid temporary postoperative symptoms. For the purpose of our study, we only included data from those who had completed both the 0 to 10 fatigue scale and MQOL-C (n = 152; 65% of all subjects).

**Instruments**

*0–10 Fatigue Scale.* A single item on the 0 to 10 numeric rating scale was used to measure CRF in the study ("0" = no fatigue, "10" = worst fatigue imaginable). The question asked about fatigue that participants had experienced in the past week (McMillan et al., 2014). On a 0 to 10 scale, they were asked how severe or intense this symptom was. Use of a single-item instrument has been suggested for routine clinical assessment and for identifying cancer patients who may significantly benefit from targeted symptom management (Butt et al., 2008).

*Multidimensional Quality of Life Scale–Cancer*. The MQOL-C was used to assess QOL (Padilla & Grant, 1985; Padilla et al., 1992). The MQOL-C has 33 items that measure 5 domains of QOL (Pinar, 2004; Pinar et al., 2003). Physical well-being (7 items) includes questions related to "present health status," "able to do things I like to do," "strength," "tire easily," "able to sleep," "able to work," and "able to get around as desired." Psychological well-being (12 items) contains "adjusting to disease or treatment," "enjoying life," "worry about financial security," "feeling useful," "feeling happy," "satisfying life," "worry about disease," "able to concentrate," "having a good quality of life," "satisfying appearance," "worry about unfinished business," and "meaningful life." Interpersonal well-being (5 items) comprises "love from others," "interference with relation," "able to fulfill responsibilities," "receive emotional support," and "make others happy." Nutrition domain (4 items) covers "appetite," "able to eat," "worry about weight," and "taste changes." Symptom distress domain (5 items) has "pain distress," "pain amount," "bowel movements," "nausea," and "vomit."

Responses are scaled from 0 to 10, with mean scores that ranged from 0 (lowest QOL) to 10 (highest QOL). Its construct validity, concurrent validity, discriminant validity, and content validity have been supported (Padilla, 1992). Factor analysis confirmed the presence of five factors in the MQOL-C (Pinar, 2004). In our study, the Cronbach’s alpha was 0.9 for the total QOL, 0.7 for physical well-being, 0.9 for psychological well-being, 0.7 for interpersonal domain, 0.7 for nutrition domain, and 0.7 for symptom distress domain. MQOL-C has been applied to differentiate among mild, moderate, and severe pain intensity subgroups in cancer patients (Paul et al., 2005). 

*Demographic and Clinical Data Form.* Demographic and clinical variables were collected by self-report. Items included age, gender, marital status, education, ethnic background, cancer diagnosis, and time after diagnosis.

**Procedures**

Following approval by the scientific review committees of the medical center and the cancer center, the project was then submitted to and received approval from the university-based Institutional Review Board. Participants were recruited from the designated oncology unit and outpatient clinic at the medical center and from the outpatient clinics at the cancer center. Patients were often approached in infusion centers in settings where they were in private bays, seated comfortably, and able to sign the informed consent document and respond to the questionnaires while receiving their treatment. The study was explained, the consent document was given to the participants, and the questions were answered. After the patient signed the consent, a research assistant administered the study questionnaires.

**Data Analyses**

To prepare the data analyses, we used the 0 to 10 fatigue scale scores to classify participants into four-level CRF subgroups based on NCCN Clinical Practice Guidelines (Berger et al., 2015): no CRF (score of 0), mild CRF (score of 1–3), moderate CRF (score of 4–6), and severe CRF (score of 7–10). The empirical evidence also supported that the optimal cutoff points were ≤ 3 for mild CRF and ≥ 7 for severe CRF among patients with and without current cancer treatment (Mendoza et al., 1999; Stover et al., 2013; Wang et al., 2014). These studies used a published criterion to identify the optimal cutoff point pair on the 0 to 10 fatigue scale (Serlin, Mendoza, Nakamura, Edwards, & Cleeland, 1995). The criterion required applying multivariate analysis of variance (MANOVA) to test various cutoff point pairs between 1 and 10 fatigue scores. The 3/7 cutoff point pair had the greatest combination of F values (i.e., Wilks lambda test, Pillai’s trace test, and Hotelling’s trace test) that demonstrated the highest discrimination among fatigue levels (Mendoza et al., 1999; Stover et al., 2013; Wang et al., 2014).

We conducted univariate analyses on demographic and clinical variables, the four-level CRF, and MQOL-C (total QOL and individual QOL domains) to generate means, standard deviations, and percentages. Histograms and cross tabulations were used to check distribution and missing values. Fifteen out of 33 MQOL-C items contained either 1 or 2 missing values. 

The research questions were answered using multiple linear regression models with post hoc analyses while adjusting for age, gender, education, marital status, racial background, cancer type, and time after diagnosis. *p* ≤ .05 was used to determine statistical significance. All analyses were performed using SAS 9.2.

Multiple linear regression analyses were performed to assess the effect of the four-level CRF (the main predictor variable) on the total QOL mean score (the dependent variable). For the five QOL domains (physical well-being, psychological well-being, interpersonal well-being, nutrition domain, and symptom distress domain), we calculated their mean scores as the dependent variables for five separate multiple linear regression analyses, with the four-level CRF as the main predictor variable. Type 3 analysis was performed to obtain the likelihood ratio test *p* value for assessing the effect of four-level CRF as a categorized predictor variable. *R²* was also extracted to assess the goodness of fit of the model. In the post hoc analyses, we conducted pairwise comparisons to identify if any between-CRF-level differences in total QOL and QOL domains existed (i.e., no vs. mild, no vs. moderate, no vs. severe, mild vs. moderate, mild vs. severe, and moderate vs. severe). Least squares means for four-level CRF subgroups were estimated and their differences were tested. Least squares means are more suitable for the unbalanced design due to missing values while adjusting for other covariates (Searle, Speed, & Milliken, 1980). 

## RESULTS

The study participants reported an average 61.25 (Standard deviation [SD] = 12.47) years of age and 14.24 (SD = 2.52) years of education (Table 1). Most of the participants were female (57.89%), currently married (61.18%), and white (82.24%). The time after diagnosis averaged 45.39% for less than 1 year, 40.13% for 1 to 5 years, and 14.48% for more than 5 years. Various types of cancer were well represented, with percentages from 9.21% (lung cancer), 10.53% (genitourinary cancer), 12.50% (breast cancer), 15.13% (other cancers), 21.71% (gastrointestinal cancer), to 30.92% (lymphoma and hematologic malignancies combined).

**Table 1:**
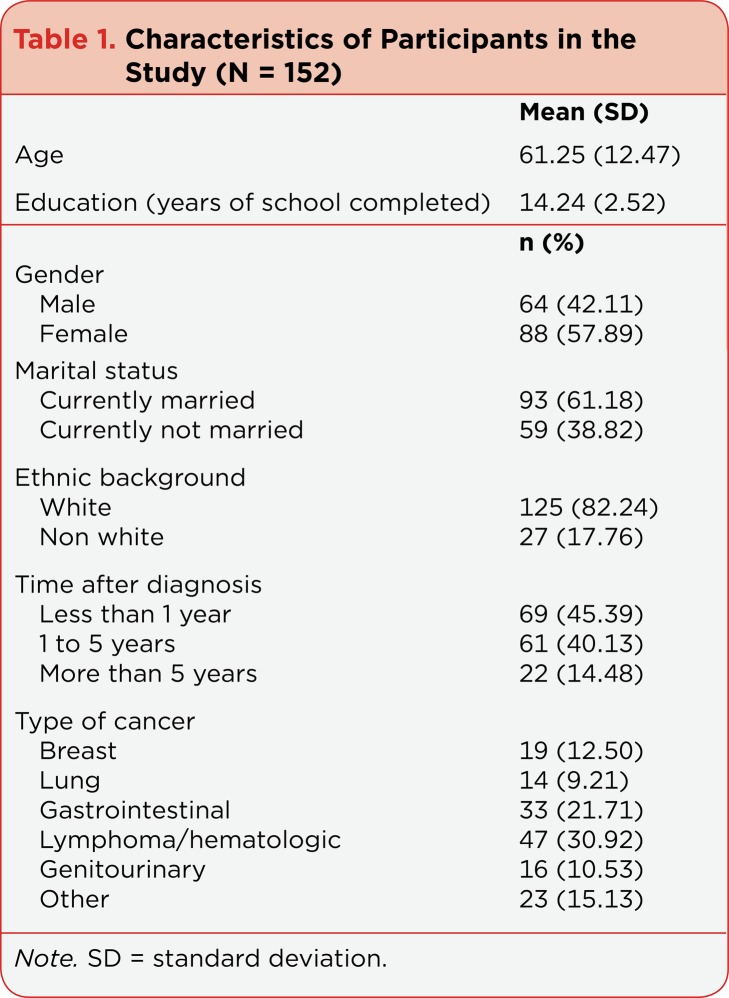
Characteristics of Participants in the Study (N = 152)

The CRF and QOL scores in the study are shown in Table 2. In the total group, CRF intensity had a mean of 4.38 (SD = 3.20). The four-level CRF showed that the majority suffered either moderate (30.26%) or severe (30.24%) CRF. The total QOL score had a mean of 6.79 (SD = 1.35). Among the QOL domains, the nutrition domain had the lowest mean score of 6.26 (SD = 2.13), while interpersonal well-being had the highest mean score of 7.84 (SD = 1.58). In the four-level CRF subgroups, the less severe the CRF level, the greater the QOL score.

**Table 2:**
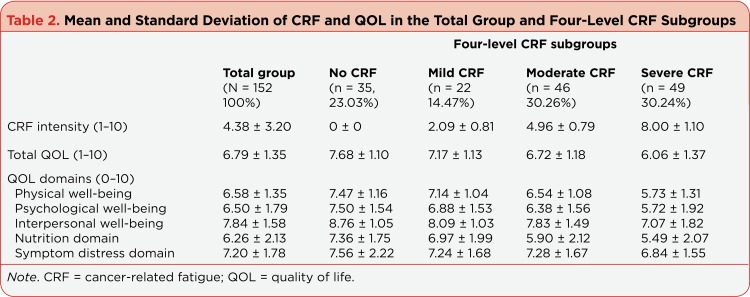
Mean and Standard Deviation of CRF and QOL in the Total Group and Four-Level CRF Subgroups

Table 3 shows the results from the multiple linear regression analyses of total QOL and QOL domains across the four CRF levels after adjusting for covariates, including age, gender, education, marital status, racial background, cancer type, and time after diagnosis. The four-level CRF was significantly associated with total QOL, physical well-being, psychological well-being, interpersonal well-being, and nutrition domain, with all type 3 *p* values less than .01. Participants in the less severe CRF subgroup had significantly better scores on total QOL (*R²* = 0.29; *p* < .01), physical well-being (*R²* = 0.34; *p* < .01), psychological well-being (*R²* = 0.22; *p* =.02), interpersonal well-being (*R²* = 0.22; *p* = .02), and nutrition domain (*R²* = 0.26; *p* < .01). No covariates were significant in these five models. On the other hand, the four-level CRF was not associated with the symptom distress domain, but older age and more years of education were significantly associated with the better symptom distress domain (*R²* = 0.16; *p* < .01). 

**Table 3:**
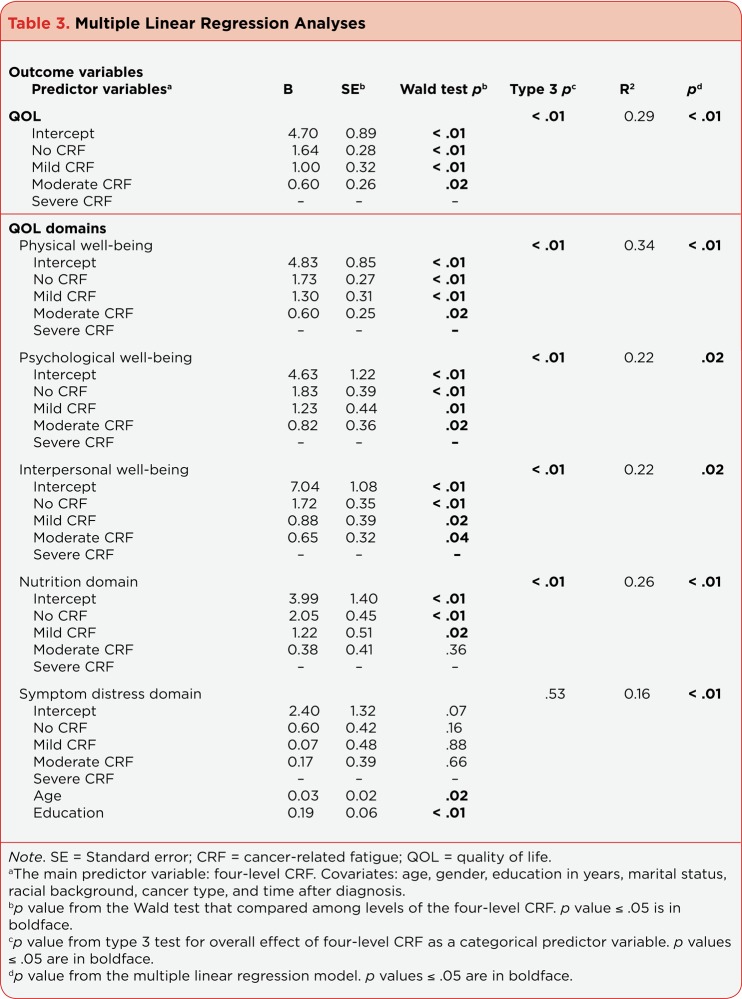
Multiple Linear Regression Analyses

Table 4 shows the least squares means of total QOL and QOL domains across the four-level CRF subgroups adjusting for age, gender, education, marital status, racial background, cancer type, and time after diagnosis. The least squares means of total QOL and QOL domains decreased while the CRF intensity went from no to mild, mild to moderate, and moderate to severe. The pattern of the between-CRF-level differences in total QOL and QOL domains varied. For the total QOL, the no CRF subgroup was significantly better than the mild CRF subgroup, and the moderate CRF subgroup was significantly better than the severe subgroup. But there was no difference between the mild and moderate CRF subgroups (total QOL: no CRF > mild CRF, moderate CRF > severe CRF).

**Table 4:**
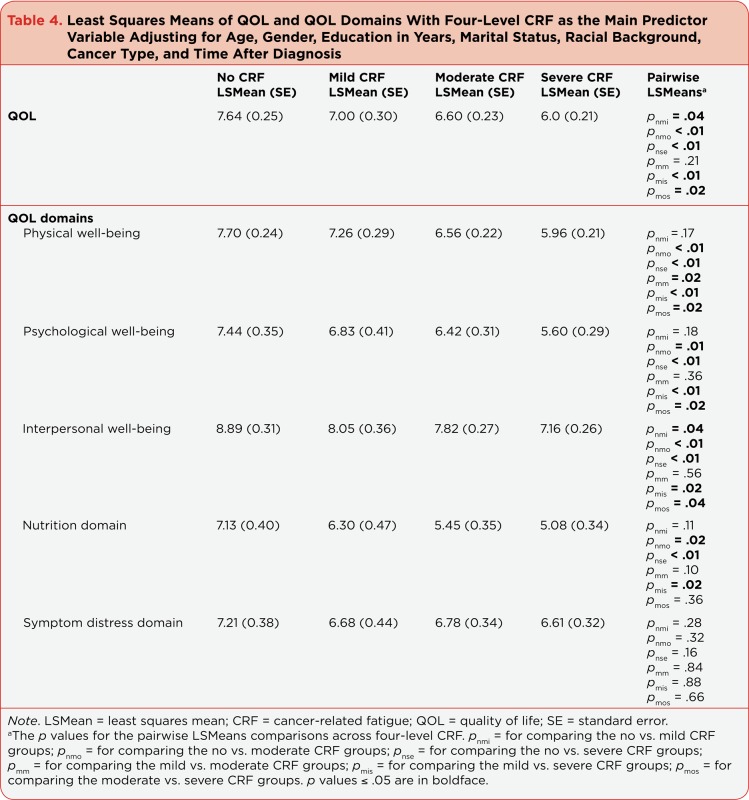
Least Squares Means of QOL and QOL Domains With Four-Level CRF as the Main Predictor Variable Adjusting for Age, Gender, Education in Years, Marital Status, Racial Background, Cancer Type, and Time After Diagnosis

In the QOL domains, interpersonal well-being had a similar pattern of between-CRF-level differences to the total QOL (interpersonal well-being: no CRF > mild CRF, moderate CRF > severe CRF). For physical well-being, the mild CRF subgroup was significantly better than the moderate CRF subgroup, and the moderate group was significantly better than the severe CRF subgroup. But there was no difference between the no and the mild CRF subgroups (physical well-being: no CRF, mild CRF > moderate CRF > severe CRF). For psychological well-being, the moderate group was significantly better than the severe CRF subgroup. But there was no difference between the no and the mild CRF subgroups or between the mild and the moderate CRF subgroups (psychological well-being: no CRF, mild CRF, moderate CRF > severe CRF). The no CRF subgroup was still significantly better than the moderate and the severe CRF subgroups.

For the nutrition domain, there was no difference between the no and the mild CRF subgroups, between the mild and the moderate CRF subgroups, and between the moderate and the severe CRF subgroups (nutrition domain: no CRF, mild CRF, moderate CRF, severe CRF). Still, three comparisons of the nutrition domain showed that the lower severity CRF subgroup was significantly better than the higher severity CRF subgroups (nutrition domain: no CRF > moderate CRF; no CRF > severe CRF; mild CRF > severe CRF). Finally, there were no statistical four-level CRF differences for the symptom distress domain. 

## DISCUSSION

**Association Between the Four-Level CRF and Total QOL and QOL Domains**

Participants who were in a lower-severity level CRF subgroup had better QOL in our study. This finding is consistent with studies among Veterans Administration Medical Center (VA) cancer patients (Hwang et al., 2002) and multisite breast cancer patients (Stover et al., 2013). We used MQOL-C as the measure of QOL, while the other two studies used FACT-G in the VA study and SF-36 in the breast cancer study. The VA study, similar to ours, recruited patients with various types of cancer. The majority of participants in our study were either in the moderate CRF or severe CRF subgroups (30.26% and 30.24%, respectively), while those in the VA study were in the no CRF (37%) and moderate CRF subgroups (36%), with fewer patients in the severe CRF subgroup (9%) (Hwang et al., 2002). Our result most likely occurred because our participants were recruited from the chemotherapy infusion center, while the majority of patients in the VA study were from an outpatient clinic and might not have had chemotherapy-induced fatigue.

Among the QOL domains, participants who were in a lower severity level CRF subgroup had better physical well-being, psychological well-being, interpersonal well-being, and nutrition domain. Similar results were seen in earlier studies when QOL was measured by FACT-G (Hwang et al., 2002) and SF36 (Stover et al., 2013). Cancer patients in the VA with lower levels of CRF reported better physical well-being, functional well-being, and emotional well-being scores in the FACT-G (Hwang et al., 2002). In breast cancer patients, a lower level CRF predicted greater physical component and mental component scores of SF-36 (Stover et al., 2013). Respectively, about 40% of the variance in the physical component score and 32% of the variance in the mental component score were explained by the four-level CRF. In our study, 34% of the variance in the physical well-being and 22% of the variance in the psychological well-being were explained by the four-level CRF. Compared with the breast cancer study (Stover et al., 2013), the lower variance in our study might have resulted from the fact that we included patients with various types of cancer and that physical and psychological well-being were two of five domains in our MQOL-C rather than only two in SF-36. 

Unexpectedly, there was no association between the four-level CRF and the symptom distress domain. The items in this domain focused on five physical symptoms: pain, nausea, vomiting, diarrhea, and constipation (Padilla, 1992; Pinar, 2004). The Memorial Symptom Assessment Scale Short Form in the VA study also included these 5 symptoms among 12 symptoms on the physical symptom distress subscale (Hwang et al., 2002). The VA study found that cancer patients with a higher severity level of CRF had worse physical symptom distress. In contrast to our study, the majority of the items in the symptom distress domain were related to gastrointestinal symptoms, and our regression model controlled for multiple covariates. Among the covariates, we found that older age and more years of education were associated with better symptom distress. That is, older participants and those with more education might manage their symptom distress better when compared with their counterparts.

**Between-CRF-Level Differences in Total QOL and QOL Domains**

As might be expected, we found that the participants in our study with a lower CRF severity level had better QOL. However, significant between-CRF-level differences (i.e., no vs. mild, mild vs. moderate, and moderate vs. severe) were only found for some of the comparisons in the total QOL and QOL domains.

For total QOL, the no CRF subgroup was better than the mild CRF subgroup; the moderate CRF subgroup was better than the severe CRF subgroup. The means of total QOL scores between the mild and moderate CRF subgroups were close, with the trend in the expected direction (mild:moderate = 7.17:6.72), so no significant difference was found. However, the mean difference of CRF scores between the mild and the moderate CRF subgroups was almost 3 points (mild:moderate = 2.09:4.96). Although the mild CRF subgroup experienced less CRF severity than the moderate CRF subgroup, their QOL was about the same. In the QOL domains, interpersonal well-being had a similar between-CRF-level difference pattern to the total QOL.

From our data and the NCCN Clinical Practice Guidelines, we suggest that clinicians still need to routinely screen for both CRF and QOL in patients with mild CRF (Berger et al., 2015). Patients with mild CRF possibly experience similar QOL reduction to those with moderate CRF. This reduction may be primarily from the interference of interpersonal well-being.

For physical well-being, participants with mild CRF were better than those with moderate CRF; participants with moderate CRF were also better than those with severe CRF. But participants with mild CRF were no different from those with no CRF. This pattern was similar to the FACT-G physical well-being scores in the VA study (Hwang et al., 2002). Significant impacts on patients’ physical well-being may happen when CRF is moderate to severe. Our findings support NCCN Clinical Practice Guidelines, which set comprehensive evaluation and specific interventions for patients with a fatigue score between 4 and 10 on a 0–10 scale (Berger et al., 2015).

For psychological well-being and nutritional well-being, patients in the lower severity CRF subgroups had better scores than those in the higher-severity CRF subgroups. However, between-CRF-level differences in the psychological well-being and nutrition domain did not show four distinct levels (i.e., no > mild > moderate > severe). It is possible that these two domains were influenced significantly by other factors that were related to CRF but not investigated in this study, such as depression (Bower, 2014) and malnutrition due to cancer or its treatment (Bozzetti et al., 2012).

## STUDY STRENGTHS AND LIMITATIONS

Our study has multiple strengths. The clear definition and classification of CRF levels is based on the NCCN Clinical Guidelines as well as strong empirical evidence (Berger et al., 2015; Bower et al., 2014; Mendoza et al., 1999; Stover et al., 2013; Wang et al., 2014). While our participants had various cancer types, their demographic and clinical factors were controlled in the statistical analyses.

But along with these strengths, there are still several limitations to the study that need to be addressed. First, we categorized a continuous measure (a 0–10 scale) into four-CRF-level subgroups. Categorization of the quantitative measure is usually not suggested in the methodologic literature (MacCallum, Zhang, Preacher, & Rucker, 2002; Royston, Altman, & Sauerbrei, 2006). However, in clinical research, classification of distinct and meaningful subgroups based on the severity of fatigue symptoms is clinically relevant (Piper & Cella, 2010). In our study, we used a well-established criterion to define CRF cutoff points and investigated whether the four-level CRF distinguished QOL and QOL domains measured by MQOL-C. The result supported the importance of using NCCN Clinical Practice Guidelines to screen, evaluate, and manage CRF.

Second, we used a single-item fatigue scale in the study. The literature showed that the single-item fatigue scale is highly correlated to multiple item instruments, such as the FACT-fatigue subscale (Butt et al., 2008). But the single item still limits the comparison with studies, including more detailed CRF assessments (Murphy et al., 2006). Third, the majority of our participants were female, currently married, white, and having high school education. Generalizability of our findings needs to be carefully weighed. Finally, we conducted regression analyses from a cross-sectional study, so the association of the four-level CRF with QOL and its domains should not be interpreted as causal.

## IMPLICATIONS FOR ADVANCED PRACTITIONERS

Perhaps the most important finding in our study is that the total QOL scores between the mild and the moderate CRF subgroups were not different; in contrast, the four-level CRF was negatively associated with the total QOL. This pattern was also found in interpersonal well-being. Therefore, clinicians should not ignore the manifestation of mild CRF at the time of diagnosis, during treatment, and after treatment (Bower et al., 2014). In addition to routine screening for patients with mild CRF, the NCCN Clinical Practice Guidelines suggest patient/family education and counseling as well as implementation of general strategies for CRF management (Berger et al., 2015). Clinicians can use printable materials from the American Cancer Society to guide the discussion with patients and their family members, such as "Fatigue in People with Cancer" (ACS, 2014). Furthermore, NCCN suggests general strategies for CRF management, including self-monitoring of CRF, energy conservation techniques, use of distraction, and finding meaning in the current situation (Berger et al., 2015; Bower et al., 2014).

Empirical evidence and clinical guidelines suggest that all patients suffering from CRF should initiate and maintain physical activity (Barsevick et al., 2013; Bower et al., 2014). Cancer patients are encouraged to engage in a moderate level of physical activity (150 minutes of aerobic activities per week) along with strength training (2 to 3 sessions per week). Clinicians need to carefully suggest the intensity, duration, and frequency of the physical activity. Patients with mild, moderate, or severe levels of CRF experienced three distinct levels of physical well-being in our study. Therefore, the design of the physical activity prescription for patients with CRF may include the four-level CRF as an indicator of tolerance for the personalized physical activity prescription.

## CONCLUSION

We set out to identify the association between clinically relevant four-level cancer-related fatigue and quality of life. Participants in this secondary data analysis completed both the 0 to 10 fatigue scale and the MQOL-C. The four-level CRF included no CRF, mild CRF, moderate CRF, and severe CRF. Multiple linear regression models and post-hoc analyses were applied while controlling for several variables. We found that participants in the less severe CRF subgroup had significantly better scores on total QOL and QOL domains, except for the symptom distress domain. Significances between CRF-level differences were only found in some of the QOL score comparisons. No difference between mild and moderate CRF subgroups was found in the total QOL or in interpersonal well-being. There was no difference between the no and the mild CRF subgroups in physical well-being. Our findings support the importance of using NCCN Clinical Practice Guidelines to screen, evaluate, and manage CRF. Advanced practitioners should be aware of mild CRF at the time of diagnosis, during treatment, and after treatment.
